# The Role of Configural Processing in Face Classification by Race: An ERP Study

**DOI:** 10.3389/fnhum.2015.00679

**Published:** 2015-12-21

**Authors:** Jing Lv, Tianyi Yan, Luyang Tao, Lun Zhao

**Affiliations:** ^1^Department of Psychology, General Hospital of People's Liberation ArmyBeijing, China; ^2^School of Life Science, Beijing Institute of TechnologyBeijing, China; ^3^Institute of Forensic Medicine, Soochow UniversitySoochow, China; ^4^School of Education, Institute of User Experience and Brain Cognition, Beijing Normal UniversityZhuhai, China; ^5^School of Psychological Research, Beijing Yiran Sunny Technology Co. LtdBeijing, China

**Keywords:** face perception, other-race faces, configural processing, ERPs

## Abstract

The current study investigated the time course of the other-race classification advantage (ORCA) in the subordinate classification of normally configured faces and distorted faces by race. Slightly distorting the face configuration delayed the categorization of own-race faces and had no conspicuous effects on other-race faces. The N170 was sensitive neither to configural distortions nor to faces' races. The P3 was enhanced for other-race than own-race faces and reduced by configural manipulation only for own-race faces. We suggest that the source of ORCA is the configural analysis applied by default while processing own-race faces.

## Introduction

It is documented that although humans can identify faces of their own race better than of other-race faces (the other-race effect, Meissner and Brigham, 2001; Sporer, 2001), the classification of faces by race is faster for other-race than own-race faces, a phenomenon labeled “the other-race classification advantage” (ORCA; Valentine and Endo, 1992; Levin, 1996, 2000; Caldara et al., 2004; Zhao and Bentin, 2008, 2011; Liu et al., 2014). Recently, we found that both Chinese and Israeli participants revealed the ORCA, whereas the observers' race did not interact with the race of the rated face either for gender or for age categorization (Zhao and Bentin, 2008). A recent behavior study showed that the ORCA was larger when the configuration of the inner-face components was distorted, reflecting delayed categorization of own-race distorted faces relative to own-race normally configured faces. These data demonstrate that one source of the ORCA in race categorization is the configural analysis applied by default while processing own-race but not other-race faces (Zhao and Bentin, 2011). The goal of the present study was to explore the time course of applying configural computations while a face's race is determined, recording Event-Related Potentials (ERPs) elicited by own- and other-race faces manipulated to attract attention to their second-order relations.

Several ERP components may index different stages in face processing. The earliest component that has been consistently associated with faces is the N170, which is a negative ERP occurring between 140 and 180 ms after stimulus onset at occipito-temporal electrodes and is reliably larger to faces than other stimulus categories (Bentin et al., 1996). It has been shown that the N170 is triggered by the detection of global face structures as well as other face related information in the visual field; it is not sensitive to configural processing^1^ (Mercure et al., 2008) but it might reflect the processing of face features (e.g., Bentin et al., 2006). In line with the insensitivity of the N170 to the face identity, several studies showed that the N170 was not sensitive to the face's race (James et al., 2001; Caldara et al., 2003, 2004; Senholzi and Ito, 2012; Sun et al., 2014); Other studies reported larger N170 for other- than own-race faces (e.g., Herrmann et al., 2007; Stahl et al., 2008, 2010; He et al., 2009; Caharel et al., 2011; Wiese, 2013; Wiese et al., 2014; see for a review, Ito and Bartholow, 2009; Senholzi and Ito, 2012) or opposite pattern (e.g., Ito and Urland, 2005; Senholzi and Ito, 2012). Indeed, the N170 differences in processing faces across races are contingent upon one's goal state (Senholzi and Ito, 2012). Particularly relevant to the present study, several studies investigated the configural processing across races by recording N170 component. In one cross-culture study with well-controlled low-level visual properties of the stimuli, both Caucasian and East Asian observers exhibited enhanced N170 inversion effect for own-race than other-race faces, indicating more configural perceptual processes (reflected by the face inversion) for own-race than other-race faces (Vizioli et al., 2010). In contrast, recent works showed similar N170 inversion effect for own and other-race faces (Wiese et al., 2009). How could these apparent discrepancies among those studies be reconciled? A possible answer comes considering the difference in the task demands (cf., Senholzi and Ito, 2012).

Interestingly, face inversion does affect many faces of configural processing (processing global information, holistic processing, and processing second-order relationship) as well as featural processing and hence, the role of the second-order configural processing in faces' race detection is not reliably revealed by N170 although it is not sensitive to second-order configural processing (Mercure et al., 2008).

Another considerable ERP component associated with categorization is occipital-temporal N250, which appears sensitive to the activation of a specific structural face representation (Schweinberger et al., 2004). Recent evidence showed that N250 is sensitive to longer term acquisition of face representations and larger for more familiar faces. For example, Tanaka et al. (2006) found that learning initially unfamiliar faces can increase the N250 component as the marker of subordinate level identification. Specifically, Tanaka and Pierce (2008) reported the increased N250 after subordinate level training that enhanced memory for other-race faces. Using repetition priming paradigm, a recent study did not find difference of N250 for repetitions of own-race vs. other-race faces but lager late N250 for unprimed own-race than other-race faces (Herrmann et al., 2007). In addition, the sensitivity of the N250 to face familiarity suggests that it should also reflect a perceptual mechanism involved in configural computations. There was evidence that the N250 was delayed by inverted vs. upright faces (Itier and Taylor, 2004) and by misaligned relative to aligned half-faces in the composite task (Letourneau and Mitchell, 2008). To date, however, no study directly examined the effect of configural processing on N250 in the task of race classification of faces.

Finally, the third ERP component that will be investigated in this study in conjunction to categorizing the race of faces, in particular to face configural processing, is the P300, a relatively late positive component with a centro-parietal of centro-frontal midline distribution (Donchin, 1981; Polich, 2007). It has been shown that the P3 amplitude is largely determined by the stimulus relevance (Gray et al., 2004), by the amount of attention allocated to the stimulus (Kok, 2001) and by the task complexity (Johnson, 1986) and that its latency reflects the length of stimulus evaluation processes (e.g., McCarthy and Donchin, 1981). Recently, converging evidence showed the racial modulation of P3, higher P3 to faces of other-race than own race (Urland and Ito, 2001; Ito and Urland, 2003, 2005; Sun et al., 2014). Importantly, there was evidence that the P3 is sensitive to facial configural computation. For example, Mercure and colleagues found that the P3 peaks earlier to configural processing during face matching according to the dimension that was task relevant (Mercure et al., 2008). To this end, our prediction was that the latency and/or the amplitude of the P3 would correlate with the speed of processing own and other-race faces, and configural processing.

In the current experiment we explored to what extent configural processes are differently applied to own-race and other race faces when face individuation is not required and, more importantly, how and where such difference can be observed along the face-processing time course. To achieve this goal we manipulated second order relations between the inner components of Chinese and Caucasian faces and recorded ERPs elicited by the normally configured and slightly distorted faces while Chinese participants classified the race of these faces. In addition, because the P1, N170, and P3 are modulated by emotional expression (e.g., Nomi et al., 2013), all images were equated for luminance and root mean square (RMS) contrast and the emotional expression in stimuli was controlled. Our working hypothesis was that distortion of second order relations within inner components should increase the time needed to extract the configural codes. Therefore, if configural computations delay race-decisions for own-race but not for other-race faces the perceivable distortions of second-order relations should augment the ORCA. Specifically, this augmentation should reflect the increase in the RTs and larger modulation for ERP components to own-race faces but have a considerably smaller effect on other-race faces.

## Methods

### Participants

The participants were 26 Chinese undergraduates from Jinan Military General Hospital in China (20 females, 20–26 years). All participants had normal or corrected to normal visual acuity and had no history of psychiatric or neurological disorders. They were right handed based on self report and were paid for participation. All participants signed an informed consent approved by the Ethical Committee of General Hospital of People's Liberation Army and were paid for their participation. One subject was excluded from the analysis due to artifacts during EEG recording.

Either before or after the electroencephalogram (EEG) recording (counterbalanced across participants), participants completed one self-report questionnaire regarding other-race contact, including five items modeled after Walker et al. (2008). Item (i) asked, “How many Caucasian people do you know very well?” with the answer choices: Up to 2, Up to 5, Up to 8, Up to 12, and More than 12. All participants reported that they do not know Caucasian people. Items 2–4 used the following scale: strongly agree (5 point), sort of agree (4 point), not sure (3 point), sort of disagree (2 point), strongly disagree (1 point) and were worded as: (i) “I often talk to Caucasian people in college,” (ii) “I often see Caucasian people outside of college,” (iii) “I often hang out with Caucasian people,” and (iv) “I often see Caucasian people at social events I attend.” Overall, almost all participants reported below mid-point social-contact, *M* = 2.3, *SD* = 0.5, indicating the participants had relatively little experience with faces from the other race.

### Stimuli^2^

**Figure 1:**
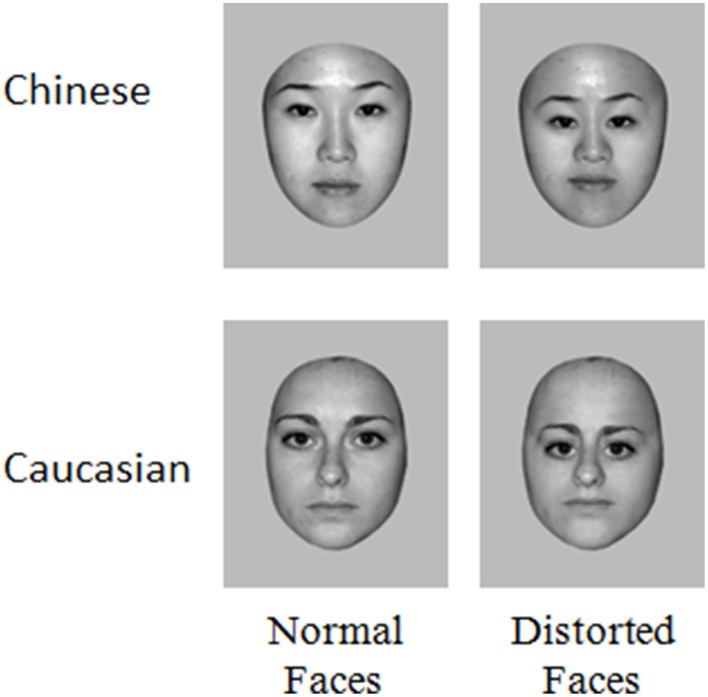
**Examples of the stimuli used in this study**.

Stimuli consisted of 70 grayscale photographs of Chinese (35 male, 35 female) and 70 Caucasian (35 male, 35 female). All faces were of young people (20–30 years old) and were unfamiliar to the participants. The distortion of second order relations in each of these faces were made by reducing the distance between the eyes by 20%, lowering the eyes' level relative to the tip of the nose by 20% and reducing the distance between the root of the nose and the mouth by 20%. All images were equated for luminance and root mean square (RMS) contrast (not including gray background in calculation) and the emotional expression in stimuli was controlled (neutral faces), using Adobe Photoshop (www.adobe.com) (Figure 1). Including background, the stimuli included 360 ^*^ 360 pixels. Seen from a distance of 70 cm they subtended a 9.9° of visual angle (~12 cm).

### Procedure

The 280 stimuli were randomized and presented at the center of a computer monitor in two blocks of 140 stimuli each separated by a short break. Each stimulus was exposed for 500 ms and followed by an inter-stimulus interval (ISI) that varied randomly between 2000 and 2500 ms. Participants were instructed to classify each stimulus by race (Chinese or Caucasian) as quickly and as accurately as possible and report their decision by pressing alternative buttons one with the index finger of the right-hand and the other with the index finger of the left hand. The assignment of the buttons to response alternatives was counterbalanced across participants.

The experiment was run in an electrically isolated and sound-attenuated booth. Following electrode montage and instructions a short training set including 12 normal configured faces and 12 distorted faces equally from each race were presented using the same procedure as in the actual experiment. None of the training stimuli were used in the experiment.

### ERP recordings

The electroencephalogram (EEG) was recorded by 64 Ag/AgCl electrodes cap according to the extended 10/20 system and continuously sampled at 512 Hz by ANT asaLab (www.ant-neuro.com). The band-pass filter range of 0.1–100 Hz was used during EEG recording at both locations. Vertical EOG and horizontal EOG were recorded with two pairs of electrodes, one placed above and below right eyes, and another 10 mm from the lateral canthi. An additional electrode as the reference electrode was placed on the tip of the nose. Throughout the EEG recording, the impedance of the electrodes was kept under 5 kΩ.

EEG data processing was performed off-line using ASA 4.9 (www.ant-neuro.com). After EOG artifacts correction, remaining artifacts exceeding ±100 μV in amplitude or containing a change of over 100 μV within a period of 50 ms were rejected at both locations. Artifact-free EEG was then segmented into epochs ranging from 200 ms (as the baseline correction) before to 800 ms after stimulus onset and averaged separately for each participant and for each of the 4 conditions Chinese normal faces, Chinese distorted faces, Caucasian normal faces, and Caucasian distorted faces. Only correct responses were included in the average but all averages included at least 50 trials. The averaged waveforms were digitally low-pass filtered at 30 Hz (24 dB/octave) to reduce high-frequency noise.

### Data analysis

Accuracy rates and RTs (from the stimulus onset) were recorded and analyzed with Race-of-the-stimulus (own-race, other-race) and Configuration (normally configured faces, distorted faces) as the within-subjects factors.

In line with most previous studies of N170 (e.g., Bentin et al., 1996), we analyzed this component at the lateral and temporo-occipital sites, P8, PO8, and P10 over the right hemi-scalp and the homologous areas over the left hemi-scalp. The peak amplitudes and latencies of the N170 component were measured between 120 and 210 ms post stimuli onset and analyzed by mixed-model ANOVA with Race-of-the-stimulus (own-race, other-race), Configuration (normally configured faces, distorted faces), Hemisphere (left, right), and Site (P7/P8, PO7/PO8, P9/P10) as the within-subjects factors. For the N250 component, since in many participants the N250 peak was not easily discernible at all sites and in each condition, this analysis was based on the mean amplitude calculated between 200 and 300 ms at same sites as N170 measures. The peak amplitudes and latencies of P3 component at midline the 4 sites (Fz, Cz, Pz, Oz) were measured between 300 and 600 ms post stimuli onset and analyzed by mixed-model ANOVA with Race-of-the-stimulus (own-race, other-race), Configuration (normally configured faces, distorted faces), and Site (Fz, Cz, Pz, Oz) as the within-subjects factors were. Degrees of freedom were corrected whenever necessary using the Greenhouse–Geisser epsilon correction factor.

## Results

### Performance

For each participant and experimental condition RTs that were more extreme than ±2 SD from the mean have been excluded (less than 2%). The ANOVA revealed significant main effects of Race-of-the-stimulus [*F*_(1, 50)_ = 71.2, MSE = 85027, *p* < 0.001; partial η^2^ = 0.59] and Configuration [*F*_(1, 50)_ = 23.7, MSE = 4495, *p* < 0.001; partial η^2^ = 0.32], reflecting that, overall, other-race faces were identified more quickly (525 ± 90 ms) than own-race faces (565 ± 83 ms), i.e., ORCA, and altering configuration of faces delayed race classification (540 ± 101 ms and 550 ± 92 ms for normal and distorted faces, respectively). Importantly, the two-way interaction of Race-of-the-stimulus ^*^ Configuration [*F*_(1, 50)_ = 37.4, MSE = 9539, *p* < 0.001; partial η^2^ = 0.43]. The *post-hoc* analysis of this two-way interaction revealed that the ORCA was significant for both normally configured faces (30 ± 18 ms; *p* = 0.018 < 0.02) and for distorted faces (59 ± 35 ms; *p* < 0.001), but it was significantly larger for the latter than for the former faces set (*p* = 0.008 < 0.01), and that the changes of facial configuration delayed race classification only for own-race faces (*p* = 0.007 < 0.01) not for other-race faces (*p* > 0.1).

For the analysis of accuracy, the other-race faces were identified more accurately (95 ± 5%) than own-race faces [92 ± 6%; *F*_(1, 50)_ = 4.1, MSE = 0.04, *p* = 0.036 < 0.05; partial η^2^ = 0.08], i.e., the ORCA of the accuracy. The main effect of Configuration was significant [*F*_(1, 50)_ = 14.4, MSE = 0.03, *p* < 0.001; partial η^2^ = 0.22] but qualified by the Race-of-the-stimulus ^*^ Configuration interaction [*F*_(1, 50)_ = 30.6, MSE = 0.065, *p* < 0.001; partial η^2^ = 0.38]. Further *post-hoc* analyses showed that the ORCA of accuracy was significant for distorted faces (89 ± 9% and 95 ± 7% for own-race and other-race faces, respectively; *p* = 0.006 < 0.01) but not for normal configured faces (94 ± 6% and 95 ± 8% for own-race and other-race faces, respectively; *p* > 0.1) and that the configuration modulation reduced the accuracy for classifying own-race faces (*p* = 0.007 < 0.01) but not for classifying other-race faces (*p* > 0.05).

### ERPs

**Figure 2:**
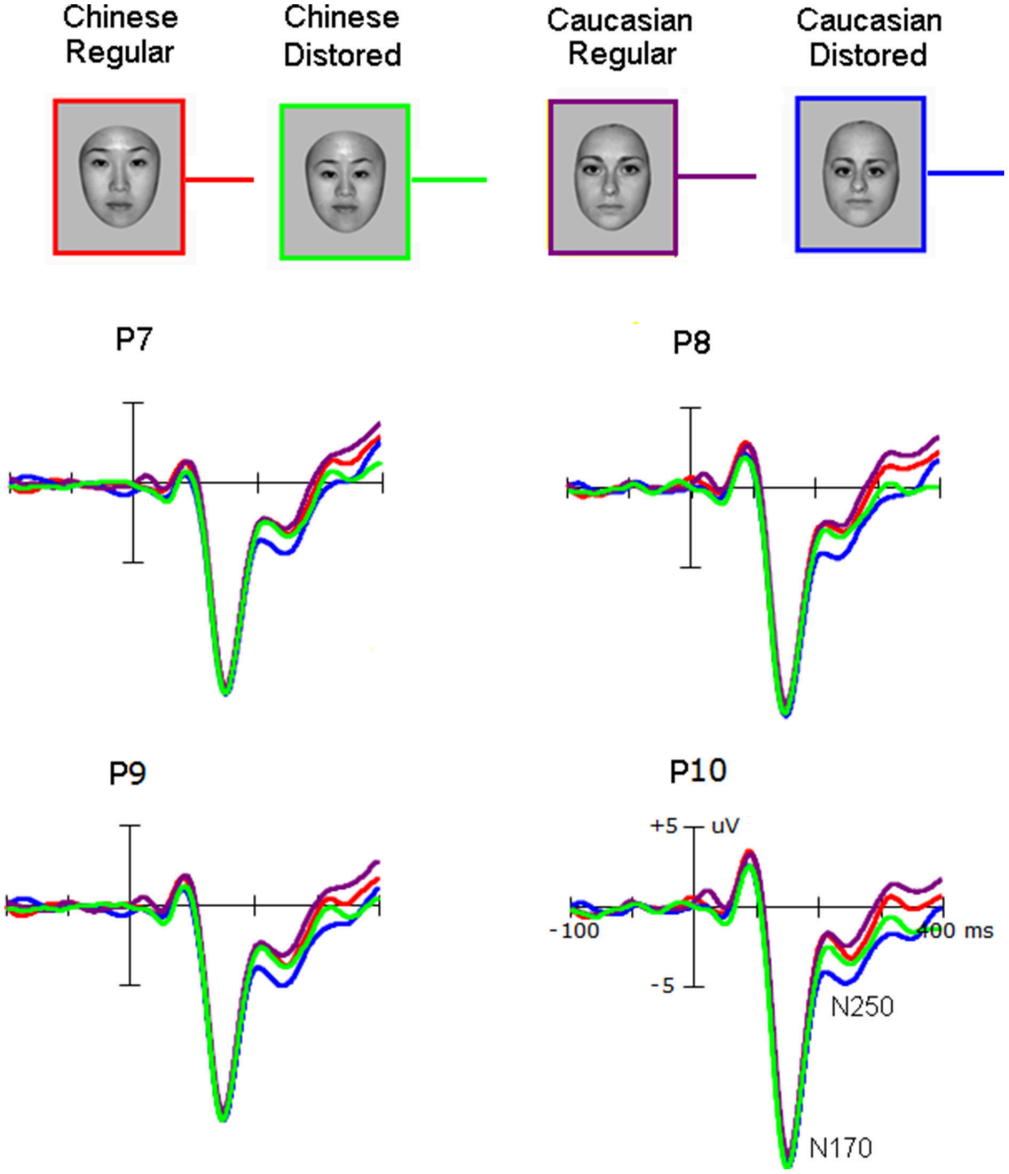
**The N170 elicited by own-race and other-race normally configured and faces with slight configural distortions**.

**Figure 3:**
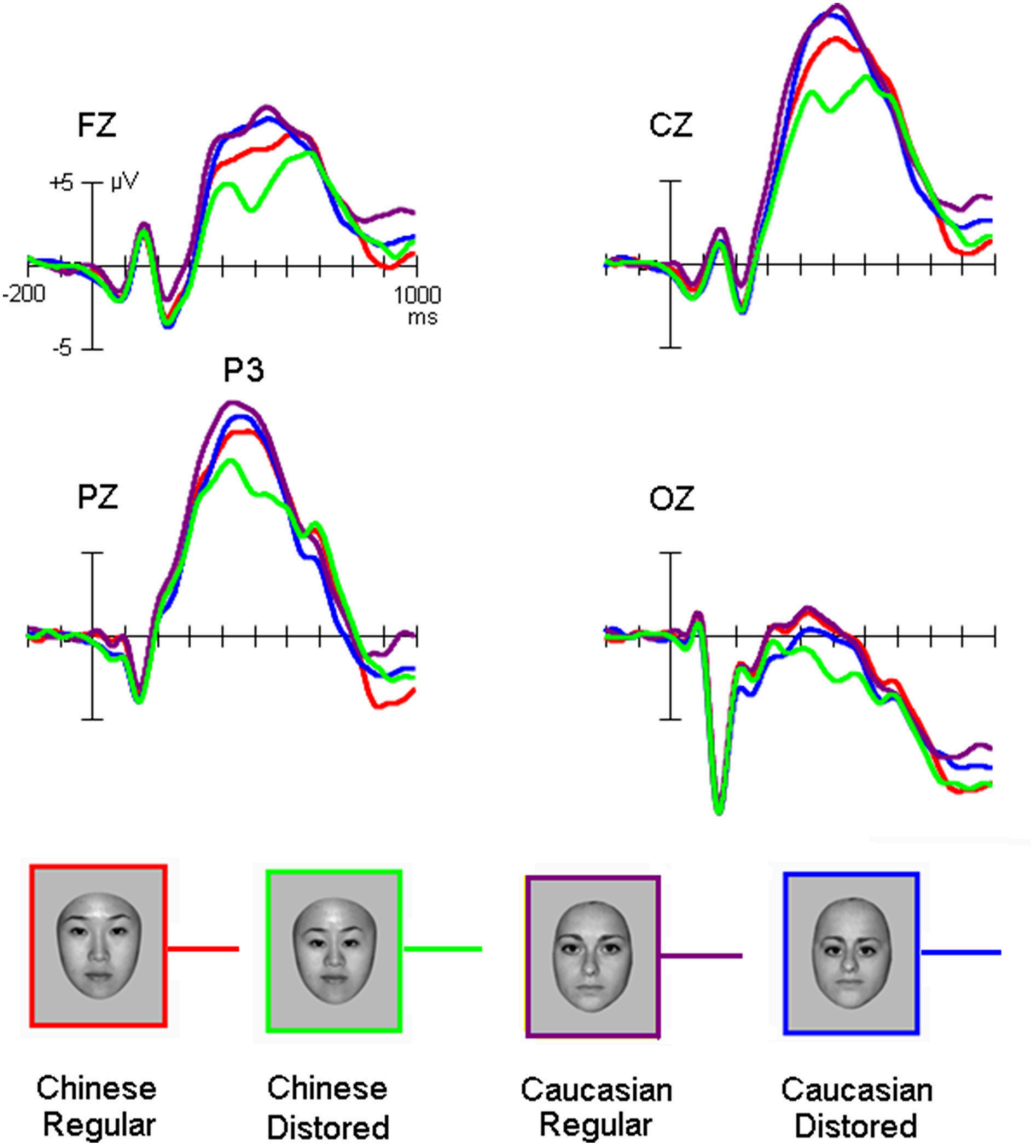
**The P3 elicited by own-race and other-race normally configured and faces with slight configural distortions**.

Grand average ERP waveforms were presented in Figures 2, 3. The salient influence of face's race occurred at the time window of the P3 component.

#### N170 component

Neither the amplitude nor the latency of the N170 was affected by the Race-of-the-stimulus (*F* < 1.0) and the Configuration (*F* < 1.0). The N170 was larger over the right hemisphere (−12.5 ± 2.8 μV) than over the left hemisphere [−10.6 ± 1.8 μV; *F*_(1, 25)_ = 17.0, *p* < 0.001; partial η^2^ = 0.40], and larger at PO7/8(−11.6 ± 2.6 μV) than at P7/8 (−10.7 ± 2.8 μV), and largest at P9/10 (−12.3 ± 3.0 μV) (*p* < 0.001; partial η^2^ = 0.26).

#### N250 component

The mean amplitudes of N250 component (200 ~ 300 ms post stimuli onset) showed no main effect of Race-of-the-stimulus (*F* < 1.0), but significant main effect of Configuration was found [*F*_(1, 25)_ = 11.488, *p* < 0.001; partial η^2^ = 0.48] and this effect was qualified by the significant interaction of Race-of-the-stimulus × Configuration [*F*_(1, 25)_ = 6.56, *p* = 0.038 < 0.05; partial η^2^ = 0.18]. *Post-hoc* analysis showed that distorting face's configuration enhanced N250 to Caucasian (*other-race*) faces [−3.3 ± −1.2 μV and −1.3 ± −0.6 μV for distorted and normal faces, respectively; *p* = 0.001] but no effect on Chinese (*own-race*) faces [−2.3 ± −1.0 μV and −1.8 ± −1.0 μV for distorted and normal faces, respectively; *p* = 0.319]. The main effect of Electrode site [*F*_(2, 50)_ = 5.045, *p* = 0.035] and interaction of Electrode site × Hemisphere [*F*_(2, 50)_ = 6.751, *p* = 0.018] were also found, reflecting significant main effect of Electrode site at left hemisphere (−2.4 ± −1.1 μV, −1.0 ± −0.3 μV and −2.9 ± −1.1 μV for P7, PO7, and P9 electrodes, respectively; *p* = 0.008) but not at right hemisphere (−2.1 ± −1.0 μV, −2.2 ± −1.0 μV and −2.5 ± −1.1 μV for P8, PO8, and P10 electrodes, respectively; *F* < 1). No other main effects and interactions were found.

#### P3 component

While the amplitude of the P3 appeared to be higher for other-race than own-race faces, the face configuration seemed to modulate the amplitude of this component for own-race more than for other-race faces (Figure 3).

ANOVA confirmed that the P3 amplitude was higher for other-race faces (13.4 ± 3.2 μV) than own-race faces [11.2 ± 2.8 μV; *F*_(1, 25)_ = 42.4, *MSE* = 929, *p* < 0.001; partial η^2^ = 0.46], that is the other-race advantage (ORA) of P3 (ORA-of-P3), and that configural distorted faces elicited smaller P3 amplitudes (11.8 ± 2.2 μV) than normally configured faces [12.8 ± 2.5 μV; *F*_(1, 25)_ = 17.5, *MSE* = 246, *p* < 0.001; partial η^2^ = 0.26]. We also found a marginally significant interaction of Configuration ^*^ Race-of-the-stimulus [*F*_(1, 25)_ = 3.6, *MSE* = 37, *p* = 0.08; partial η^2^ = 0.08] and *post-hoc* analysis revealed that whereas for own-race faces the amplitude of the P3 was larger for normally configured (12.2 ± 2.8 μV) than distorted faces (10.7 ± 3.0 μV; *p* = 0.008 < 0.01), no such effect was found for other-race faces (13.9 ± 2.8 μV and 13.2 ± 3.1 μV for normally configured and distorted faces, respectively; *p* > 0.05) and that the ORA-of-P3 was larger for distorted (3.8 ± 1.8 μV) than normal faces (1.5 ± 1.6 μV; *p* = 0.006 < 0.01). The Site effect was also significant [9.8 ± 2.2 μV, 15.5 ± 2.4 μV, 14.0 ± 2.3 μV, and 2.3 ± 2.0 μV at Fz, Cz, Pz, and Oz, respectively; *F*_(3, 75)_ = 40.7, *MSE* = 1835, *p* < 0.001; partial η^2^ = 0.45].

**Figure 4:**
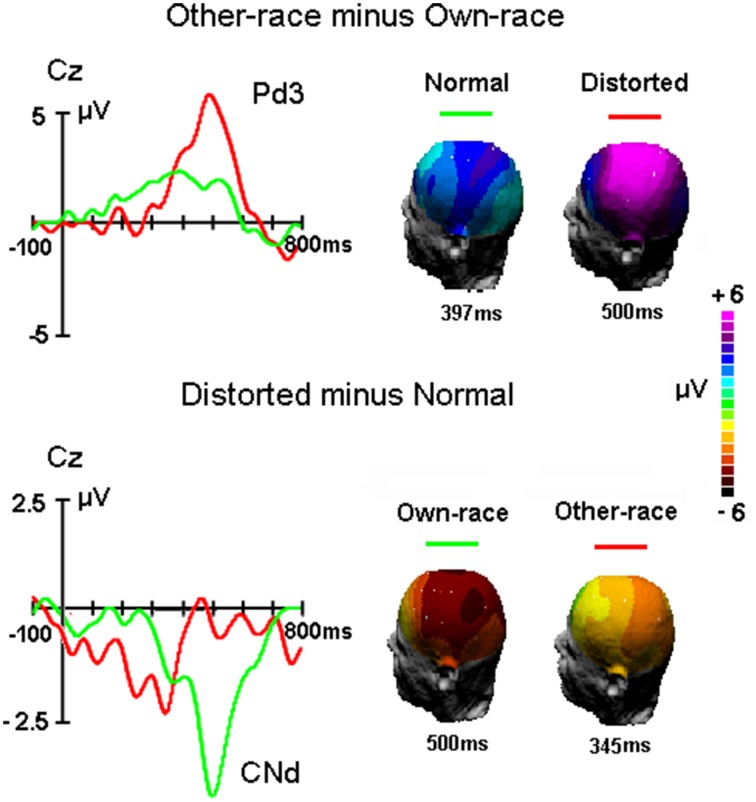
**The difference waveforms between other-race and own-race faces and between distorted and normal faces as well as the 3D mapping of each component, respectively**.

For P3 latency, there was a significant interaction of Configuration × Race-of-the-face [*F*_(1, 50)_ = 5.5, *MSE* = 16330, *p* = 0.028 < 0.03; partial η^2^ = 0.1], which suggested that whereas for own-race faces the P3 latency for distorted faces was shorter (482 ± 48 ms) than for normally configured faces (502 ± 60 ms; *p* = 0.018 < 0.02), there was no such difference for other-race faces (481 ± 56 ms and 485 ± 66 ms for normal and distorted faces, respectively; *p* > 0.1). Note that the measurement of the P3 latency elicited by distorted own-race faces might also reflect a negative-going potential that interrupted the P3 (see Figure 4).

In addition to the ANOVAs, we calculated the Person correlations between the RTs and the peak latencies/amplitudes of the N170 and P3 components. Significant negative correlations were found between the RTs and the amplitude of P3 (*r* = −0.62, *p* < 0.01) not between the RTs and the amplitude of N170 component (*p* > 0.1), that is, the longer the RTs the lower the P3 amplitude. The correlations between the RTs and the latencies of ERP components were not found (*ps* > 0.1).

#### Mean amplitudes during P3 time window in the difference waveforms

Figure 4 showed the difference waveforms by subtracting the ERP waveforms to own-race stimuli from that to other-race stimuli as well as by subtracting the ERPs to normal faces from that to distorted faces. It appears that the Pd3 component reflecting the ORA-of-P3 was modulated by altering the configuration of faces and that the CNd component (peaked around 500 ms post stimulus onset) reflecting configural processing was influenced by face's race. Overall, Pd3 was bigger for distorted than normal faces and Conf_Nd was bigger for own-race than other-race faces. To further examine the phenomena within and across races, the mean amplitudes of Pd3 as well as CNd were measured and analyzed.

For Pd3 component, mean amplitude between 450 and 550 ms was analyzed by a Two-way ANOVA with Configuration (normal, distorted) and Site (Fz, Cz, Pz, Oz) as within-subjects factors. ANOVA showed that larger Pd3 was found for distorted faces (3.8 ± 1.6 μV) than original faces [1.5 ± 0.8 μV, *F*_(1, 50)_ = 18.2, *MSE* = 507, *p* = 0.007 < 0.01]. The main effect of Site was also significant [*F*_(3, 150)_ = 10.9, *MSE* = 43, *p* < 0.001], reflecting the maximum of Pd3 at Cz site (3.2 ± 1.8 μV), i.e., central-parietal distribution as presented in Figure 4.

For CNd component, mean amplitude between 450 and 550 ms was analyzed by a Two-way ANOVA with Race-of-the-stimulus (own-race, other-race) and Site (Fz, Cz, Pz, Oz) as within-subjects factors. The ANOVA showed that the CNd component was more negative for own-race (−2.4±0.8 μV) than other-race faces (−0.2 ± 0.9 μV), *F*_(1, 50)_ = 18.2, *MSE* = 508, *p* < 0.001; partial η^2^ = 0.3.

## Discussion

In the current experiment we investigated the involvement of configural computations while processing own-race and other-race faces in a race categorization task. Analyzing performance, we found that in Chinese participants the classification of other-race faces was faster than the classification of own-race faces (ORCA). Importantly, however, the ORCA on the classification speed was twice as big for distorted faces than for normally configured faces and the second-order configural modulation reduced the performance for own-race faces not for other-race faces. Because the configural computation was task-irrelevant in the present study, these data indicated that one of the sources of ORCA is the configural analysis applied by default while processing own-race faces, replicating our previous behavioral study (Zhao and Bentin, 2011). The time course of the face-classification processes reflected in the ERP analysis also suggests a more complex pattern than assuming that the observed enhancement of the ORCA for distorted faces was a simple and straightforward result of faster decision processes for distorted faces.

Replicating recent findings (e.g., Sun et al., 2014), neither the amplitude not the latency of the N170 were modulated by the race of the face. Indeed, this pattern is in line with previous studies showing the robustness of this component to configural distortions (e.g., Zion-Golumbic and Bentin, 2007; Liu et al., 2014). Importantly, the N170 in the present study was insensitive to subtle distortions in the face configuration either for own-race or other-race faces. However, Vizioli and colleague found that both Caucasian and East Asian observers exhibited enhanced N170 inversion effect for own-race than other-race faces although face's race did not affect the N170 elicited by upright faces (Vizioli et al., 2010). It should be noticed that inversion of a face interferes with many types of configural processing, the first-order relations, holistic processes and the second-order relations, even with featural processing. Therefore, the differential configural computation could account for the divergence between Vizioli et al. findings and ours. On the other hand, there was evidence that the N170 differences in processing faces across races are contingent upon one's goal state (Senholzi and Ito, 2012) and hence the different task demands, i.e., passive detection paradigm in Vizioli et al. and active race categorization task in the present study could be another reason for distinct N170 patterns. In a word, along with studies in which regardless of task the N170 was not affected by the race of the face (e.g., Caldara et al., 2003, 2004; Tanaka and Pierce, 2009; Sun et al., 2014), the present data suggest that the subordinate categorization of faces by race follows the early processing characteristic to faces, which is reflected by the N170 component (see also, Liu et al., 2014).

In contrast to previous finding of race modulation of N250 component, we did not find race modulation on the N250 elicited by normal configuraled faces. Recent evidence showed that the posterior N250 is sensitive to two types of familiarity effects in face processing, reflecting the activation of previously stored face representations (e.g., own face, famous faces) and the plasticity of an acquired familiarity that accrues gradually over time, respectively (Tanaka et al., 2006; Tanaka and Pierce, 2008). Based on more experience that adults have for own-race than other-race faces, which could be expected is larger N250 elicited by own-race than other-race faces. This did not happen, however, in the current experiment. Instead, only for distorted condition the other-race faces elicited larger N250 than did own-race faces. Actually, the mixed N250 results are not surprising due to different task demands between previous studies and the present classification task as well as that faces used in this study were unfamiliar to all the participants. Obviously, this issue of race modulation on the N250 component awaits further investigation.

A clear pattern of effects is evident in the analysis of the P3 component. The effects of the Race-of-the-stimulus and of Configuration on the amplitude of the P3 resembled those found in performance (indeed, a significant correlation was found between RTs and the P3 amplitude across conditions and within each condition). In line with recent findings using the same race classification task (e.g., Sun et al., 2014; Liu et al., 2014), other-race faces elicited larger P3 amplitudes than own-race faces, putatively reflecting a smaller jitter in the time at which the characteristics of other-race have been recognized in individual trials. More importantly, configural distortion did not affect other-race faces while reducing the amplitude of own-race faces. Informal scrutiny of the P3 suggests that the classification of own-race faces was disturbed by configural distortion, resulting in a wider component, as presented by CNd component reflecting the configural processing of faces. Due to the task-irrelevant of configural processing in the present experiment, the CNd component could be associated with automatically analyzing configural properties of own-race faces. This ERP pattern was inconsistent with one previous report, in which incongruent configural completions (e.g., realigning features into a different arrangement) elicited a late positive component (Bobes et al., 2007) not a negative component like the present CNd component. This difference might be due to different task demands: face identification processes in Bobes et al.'s study and face classification in our study. Possibly, it is the automatic configural processing appeared at evaluation/decision level of face classification that interfere the race classification of distorted own-race faces. The absence of CNd for other-race faces (i.e., similar P3 for distorted and normal faces) provided electrophysiological evidence for behavioral finding that no negative influence of configuration was found for classifying other-race faces.

According to the above perspective based on the difference waveforms, interestingly, for Caucasian (other-race) faces, the configural effect was indeed appeared the earlier time window (200 ~ 400 ms) mainly relevant to the deflection of the N250 component. One possible explanation for configural effect for other-race faces is that participants may detect the configural changes of other-race faces at earlier stage of face identification by semantic and/or individual information (Schweinberger et al., 2004). However, that could not be the case due to the more configural processing for own-race than other-race faces (e.g., Michel et al., 2006, 2010). Alternative explanation is that the contracted other-race faces look like more homogenous than other-race faces with normal configuration (as the present behavioral results) and attract more attentional resources, and result in a faster classification by race. Indeed, supporting this explanation, in attentional studies the posterior N2 indexed the early detection of target stimuli (e.g., Potts and Tucker, 2001). More widely distribution of CNd for own-race faces than that for other-race faces also showed different mechanism of processing the configuration of faces within and across races. Moreover, it would be important to test a Caucasian group of participants to understand which of these above effects are really driven by own- and other-race faces vs. some aspects of the stimuli unrelated to face race.

Interestingly, the modulation of the P3 latency by distortion was more equivocal. The significant delay of the P3 should be cautiously interpreted given the multi-peak morphology of this component, which imposed uncertainty in the measurement of the peak latencies of the P3 elicited by own-race, configural-distorted faces. Adopting the view that the latency of the P3 is a measure of the stimulus evaluation time, this pattern suggests that the effect of the face configuration on the P3 component does not simply reflect a delay in classifying the race of own-race distorted faces but, perhaps, a different evaluation process. Indeed, as mentioned above, the measurement of the P3 latency elicited by distorted own-race faces (especially in the Chinese group) might reflect a negative-going potential (i.e., CNd) that interrupted the P3 (see Figure 4).

In sum, although Chinese participants were tested only in the present study, along with the similar interaction found for RTs, the fact that the effect of configuration of P3 was significant for own-race faces but not for other-race faces implicates that the ORCA is caused by the default tendency to process own-race faces to the individual level whereas other race faces are initially perceived at the race-group level, at least for Chinese participants. In other word, during the explicit face classification by race, other race faces are perceived as a group, whereas the grouping of own-race faces is interfered with and delayed by the automatic application of configural computations that are necessary for distinguishing among individual faces but probably irrelevant for race categorization. Due to the fact that Chinese participants were recruited only in the present study, a cross-culture research, i.e., two race groups of observers at least, would be necessary to clarify the present conclusion in the future.

### Conflict of interest statement

The authors declare that the research was conducted in the absence of any commercial or financial relationships that could be construed as a potential conflict of interest. The Review Editor Lucy Troup declares that, despite being affiliated with the same institution as the Associate Editor Carol Seger, the review process was handled objectively.

## Footnotes

^1^To avoid the confusion, the configural processing here means the process of second-order relations of faces.

^2^In our previous study (Zhao and Bentin, 2011), these stimuli had been used to compare the ability of each ethnic group to discriminate own-race and other race distorted faces from the normally configured faces and we found that both the Chinese and the Caucasian detected distorted faces significantly above chance (Chinese: *t*_(13)_ = 7.3, *p* < 0.001 and *t*_(13)_ = 8.3, *p* < 0.001 for Chinese and Caucasian faces, respectively; Caucasian: *t*_(13)_ = 6.2, *p* < 0.001 and *t*_(13)_ = 9.9, *p* < 0.001 for Chinese and Caucasian faces, respectively).

## References

[B1] Bentin S. Allison T. Puce A. Perez E. McCarthy G. (1996). Electrophysiological studies of face perception in humans. J. Cogn. Neurosci. 8, 551–565. 10.1162/jocn.1996.8.6.551 20740065 PMC2927138

[B2] Bentin S. Golland Y. Flevaris A. Robertson L. C. Moscovitch M. (2006). Processing the trees and the forest during initial stages of face perception: electrophysiological evidence. J. Cogn. Neurosci. 18, 1406–1421. 10.1162/jocn.2006.18.8.1406 16859424

[B3] Bobes M. A. Quinonez I. Perez J. Leon I. Valdes-Sosa M. (2007). Brain potentials reflect access to visual and emotional memories for faces. Biol. Psychol. 75, 146–153. 10.1016/j.biopsycho.2007.01.006 17350154

[B4] Caharel S. Montalan B. Fromager E. Bernard C. Lalonde R. Mohamed R. (2011). Other-race and inversion effects during the structural encoding stage of face processing in a race categorization task: an event-related brain potential study. Int. J. Psychophysiol. 79, 266–271. 10.1016/j.ijpsycho.2010.10.018 21055428

[B5] Caldara R. Rossion B. Bovet P. Hauert C. A. (2004). Event-related potentials and time course of the ‘other-race’ face classification advantage. Neuroreport 15, 905–910. 10.1097/00001756-200404090-00034 15073540

[B6] Caldara R. Thut G. Servoir P. Michel C. M. Bovet P. Renault B. (2003). Face versus non-face object perception and the ‘other-race’ effect: a spatio-tempORCAl event-related potential study. Clin. Neurophysiol. 114, 515–528. 10.1016/S1388-2457(02)00407-8 12705432

[B7] Donchin E. (1981). Surprise…Surprise. Psychophysiology 18, 493–513. 10.1111/j.1469-8986.1981.tb01815.x 7280146

[B8] Gray H. M. Ambady N. Lowenthal W. T. Deldin P. (2004). P300 as an index of attention to self-relevant stimuli. J. Exp. Soc. Psychol. 40, 216–224. 10.1016/S0022-1031(03)00092-1

[B9] He Y. Johnson M. K. Dovidio J. F. McCarthy G. (2009). The relation between race-related implicit associations and scalp-recorded neural activity evoked by faces from different races. Soc. Neurosci. 26, 1–17. 10.1080/17470910902949184 PMC2755624 19562628

[B10] Herrmann M. J. Schreppel T. Jager D. Koehler S. Ehlis A. C. Fallgatter A. J. (2007). The other-race effect for face perception: an event-related potential study. J. Neural Transm. 114, 951–957. 10.1007/s00702-007-0624-9 17318308

[B11] Itier R. J. Taylor M. J. (2004). Effects of repetition learning on upright, inverted and contrast-reversed face processing using ERPs. Neuroimage 21, 1518–1532. 10.1016/j.neuroimage.2003.12.016 15050576

[B12] Ito T. A. Bartholow B. D. (2009). The neural correlates of race. Trends Cogn. Sci. 13, 524–531. 10.1016/j.tics.2009.10.002 19896410 PMC2796452

[B13] Ito T. A. Urland G. R. (2003). Race and gender on the brain: electrocortical measures of attention to the race and gender of multiply categorizable individuals. J. Pers. Soc. Psychol. 85, 616–626. 10.1037/0022-3514.85.4.616 14561116

[B14] Ito T. A. Urland G. R. (2005). The influence of processing objectives on the perception of faces: an ERP study of race and gender perception. Cogn. Affect. Behav. Neurosci. 5, 21–36. 10.3758/CABN.5.1.21 15913005

[B15] James M. S. Johnstone S. J. Hayward W. G. (2001). Event-related potentials, configural encoding, and feature-based encoding in face recognition. J. Psychophysiol. 15, 275–285. 10.1027/0269-8803.15.4.275

[B16] Johnson R. (1986). A triarchic model of P300 amplitude. Psychophysiology 23, 367–384. 10.1111/j.1469-8986.1986.tb00649.x 3774922

[B17] Kok A. (2001). On the utility of P3 amplitude as a measure of processing capacity. Psychophysiology 38, 557–577. 10.1017/S0048577201990559 11352145

[B18] Letourneau S. M. Mitchell T. V. (2008). Behavioral and ERP measures of holistic face processing in a composite task. Brain Cogn. 67, 234–245. 10.1016/j.bandc.2008.01.007 18336979

[B19] Levin D. T. (1996). Classifying faces by race: the structure of face category. J. Exp. Psychol. Learn. Mem. Cogn. 22, 1364–1382. 10.1037/0278-7393.22.6.1364

[B20] Levin D. T. (2000). Race as a visual feature: using visual search and perceptual discrimination tasks to understand face categories and the cross-race recognition. J. Exp. Psychol. Gen. 129, 559–574. 10.1037/0096-3445.129.4.559 11142869

[B21] Liu Z. Zhang Q. Li Y. Du Y. Dong W. Zhao L. (2014). The role of featural processing in other-race face classification advantage: an ERP study. J. Integr. Neurosci. 3, 1–12. 10.1142/s0219635214500125 25164358

[B22] McCarthy G. Donchin E. (1981). A metric for thought: a comparison of P300 latency and reaction-time. Science 211, 77–80. 10.1126/science.7444452 7444452

[B23] Meissner C. A. Brigham J. C. (2001). Thirty years of investigating the own-race bias in memory for faces - A meta-analytic review. Psychol. Public Policy Law 7, 3–35. 10.1037/1076-8971.7.1.3

[B24] Mercure E. Dick F. Johnson M. H. (2008). Featural and configural face processing differentially modulate ERP components. Brain Res. 1239, 162–170. 10.1016/j.brainres.2008.07.098 18722354

[B25] Michel C. Caldara R. Rossion B. (2006). Same-race faces are perceived more holistically than other-race faces. Vis. Cogn. 14, 55–73 10.1080/13506280500158761

[B26] Michel C. Corneille O. Rossion B. (2010). Holistic face encoding is modulated by perceived face race: evidence from perceptual adaptation. Vis. Cogn. 18, 434–455. 10.1080/13506280902819697

[B27] Nomi J. S. Rhodes M. G. Cleary A. M. (2013). Emotional facial expressions differentially influence predictions and performance for face recognition. Cogn. Emot. 27, 141–149. 10.1080/02699931.2012.679917 22712473

[B28] Polich J. (2007). Updating p300: an integrative theory of P3a and P3b. Clin. Neurophysiol. 118, 2128–2148. 10.1016/j.clinph.2007.04.019 17573239 PMC2715154

[B29] Potts G. F. Tucker D. M. (2001). Frontal evaluation and posterior representation in target detection. Brain Res. Cogn. Brain Res. 11, 147–156. 10.1016/S0926-6410(00)00075-6 11240117

[B30] Schweinberger S. R. Huddy V. Burton A. M. (2004). N250r: a face-selective brain response to stimulus repetitions. Neuroreport 15, 1501–1505. 10.1097/01.wnr.0000131675.00319.42 15194883

[B31] Senholzi K. B. Ito T. A. (2012). Structural face encoding: how task affects the N170's sensitivity to race. Soc. Cogn. Affect. Neurosci. 8, 937–942. 10.1093/scan/nss091 22956666 PMC3831558

[B32] Sporer S. L. (2001). Recognizing faces of other ethnic groups - An integration of theories. Psychol. Public Policy Law 7, 36–97. 10.1037/1076-8971.7.1.36

[B33] Stahl J. Wiese H. Schweinberger S. R. (2008). Expertise and own-race bias in face processing: an event-related potential study. Neuroreport 19, 583–587. 10.1097/WNR.0b013e3282f97b4d 18388743

[B34] Stahl J. Wiese H. Schweinberger S. R. (2010). Learning task affects ERP-correlates of the own-race bias, but not recognition memory. Neuropsychologia 48, 2027–2040. 10.1016/j.neuropsychologia.2010.03.024 20362599

[B35] Sun G. Zhang G. Yang Y. Bentin S. Zhao L. (2014). Mapping the time course of other-race face classification advantage: a cross-race ERP study. Brain Topogr. 27, 663–671. 10.1007/s10548-013-0348-0 24375283

[B36] Tanaka J. W. Curran T. Porterfield A. L. Collins D. (2006). Activation of preexisting and acquired face representations: the N250 event-related potential as an index of face familiarity. J. Cogn. Neurosci. 18, 1488–1497. 10.1162/jocn.2006.18.9.1488 16989550

[B37] Tanaka J. W. Pierce L. J. (2008). The neural and behavioral plasticity of other-race face recognition. J. Vis. 8, 534–534. 10.1167/8.6.534 19246333

[B38] Tanaka J. W. Pierce L. J. (2009). The neural plasticity of other-race face recognition. Cogn. Affect. Behav. Neurosci. 9, 122–131. 10.3758/CABN.9.1.122 19246333

[B39] Urland G. R. Ito T. A. (2001). P300 and implicit and explicit categorization of race and gender. Psychophysiology 38, S96–S96.

[B40] Valentine T. Endo M. (1992). Towards an examplar model of face processing - The effects of race and distinctiveness. Q. J. Exp. Psychol. A Hum. Exp. Psychol. 44, 671–703. 10.1080/14640749208401305 1615169

[B41] Vizioli L. Rousselet G. A. Caldara R. (2010). Neural repetition suppression to identity is abolished by other-race faces. Proc. Natl. Acad. Sci. USA. 107, 20081–20086. 10.1073/pnas.1005751107 21041643 PMC2993371

[B42] Walker P. M. Silvert L. Hewstone M. Nobre, A. C. (2008). Social contact and other-race face processing in the human brain. Soc. Cogn. Affect. Neurosci. 3, 16–25. 10.1093/scan/nsm035 19015091 PMC2569819

[B43] Wiese H. (2013). Do neural correlates of face expertise vary with task demands? Event-related potential correlates of own- and other-race face inversion. Front. Hum. Neurosci. 7:898. 10.3389/fnhum.2013.00898 24399955 PMC3870922

[B44] Wiese H. Kaufmann J. M. Schweinberger S. R. (2014). The neural signature of the own-race bias: evidence from event-related potentials. Cereb. Cortex 24, 826–835. 10.1093/cercor/bhs369 23172775

[B45] Wiese H. Stahl J. Schweinberger, S. R. (2009). Configural processing of other-race faces is delayed but not decreased. Biol. Psychol. 81, 103–109. 10.1016/j.biopsycho.2009.03.002 19428974

[B46] Zhao L. Bentin S. (2008). Own- and other-race categorization of faces by race, gender and age. Psychon. Bull. Rev. 15, 1093–1099. 10.3758/PBR.15.6.1093 19001573

[B47] Zhao L. Bentin S. (2011). The role of features and configural processing in face-race classification. Vision Res. 51, 2462–2470. 10.1016/j.visres.2011.10.001 22008980 PMC3225710

[B48] Zion-Golumbic E. Bentin S. (2007). Dissociated neural mechanisms for face detection and configural encoding: evidence from N170 and induced gamma-band oscillation effects. Cereb. Cortex 17, 1741–1749. 10.1093/cercor/bhl100 17062635

